# Chyle and Bile Leak Following Open Cholecystectomy: A Rare Complication

**DOI:** 10.7759/cureus.59338

**Published:** 2024-04-30

**Authors:** Raneem Alzaghran, Faisal S AlGhamdi, Fahad Alshubaily, Essam Alhothaifi

**Affiliations:** 1 General Surgery Department, King Saud Medical City, Riyadh, SAU; 2 General Surgery Department, Prince Sultan Military Medical City, Riyadh, SAU; 3 Hepato-Pancreato-Biliary Surgery, General Surgery Department, King Saud Medical City, Riyadh, SAU

**Keywords:** gall stones, cholelithiasis, chronic cholecystitis, open cholecystectomy, bile leak, chyle leak

## Abstract

Gallstone disease is extremely common and frequently and safely treated by cholecystectomy. Chyle leak is a rare but significant side effect of many abdominal surgeries with rarely reported post-cholecystectomy. In this case, we report a 78-year-old lady with multiple comorbidities and symptomatic gallstones who underwent open cholecystectomy complicated by bile and chyle leak, which was successfully managed with endoscopic retrograde cholangiopancreatography (ERCP) and stenting for bile leak and conservative management for the chyle leak, which included drainage, low-fat diet, and octreotide.

## Introduction

Gallstone disease is extremely common and is frequently and safely treated by cholecystectomy all over the world. Laparoscopic surgery has been found to provide advantages such as less pain, shorter recovery time, less operating stress, and a limited inflammatory response [1]. Despite these advantages, open cholecystectomy still has been done for difficult procedures or with elderly patients with multiple comorbidities, especially cardiac diseases where laparoscopic insufflation might compromise the patient's hemodynamics. According to data from population-based research, open cholecystectomy is still performed on 21% to 55% of elderly patients in the United States [2-5].

Considering this, the surgical community suggests an operational strategy of defense against this patient group, which frequently presents with empyema, gallbladder hydrops, and acute or chronic recurrent cholecystitis. In the elderly, the severity of cholecystitis, poor physical condition, and multiple comorbidities increase the risk of complications following cholecystectomy [6]. Chylous ascites or a chyle leak is a rare but significant side effect of many abdominal surgeries. We present a rare case of chyle and bile leaks that occurred post-open cholecystectomy for chronic cholecystitis and its successful management.

## Case presentation

This was a 78-year-old woman with multiple comorbidities; bronchial asthma, pulmonary hypertension, interstitial lung disease, hypertension, and a history of atrial fibrillation, who presented with a one-year history of episodes of right upper quadrant pain associated with fatty meals radiating to right shoulder and back. She had a history of ascending cholangitis one year prior to presentation where endoscopic retrograde cholangiopancreatography (ERCP) was done with a common bile duct plastic stent placed, which had migrated and passed with her bowel motion a few months after that. On examination, all observations were within normal limits. Her abdomen was mildly tender in the right upper quadrant without any palpable masses. Laboratory investigations revealed white blood cells of 8.95 × 10^9^/l (normal, 4-11 × 10^9^/l). All liver function tests were normal. An ultrasound scan revealed a mildly enlarged fatty liver with multiple gallbladder stones without inflammatory changes; the common bile duct is not dilated.

After the patient was prepared for the surgery, her apixaban was held the day before the surgery, and her hypertension was controlled, she was taken to the operation theater and underwent open cholecystectomy with intra-op cholangiogram. The intraoperative finding was an intra-hepatic inflamed gall bladder with adhesions around it. The dissection was done using the fundus-first technique. During dissection, a lot of blood oozing from dissection planes was encountered, as the patient was on apixaban, and there was uncontrolled hypertension intraoperatively. In addition to that, after the dissection of the gall bladder from the liver bed, there was a suspected small duct of Luschka on the liver bed, which was not clearly identified later due to the use of electrocautery to control the bleeding from the liver bed. The cystic duct was short, dilated, and low-laying, which was opened, and an intra-op cholangiogram was done, which was clear. Hence, the cystic duct was then closed using 5-0 PDS interrupted stitches. After that, white gauze was applied to the gall bladder bed to check for a bile leak, and was negative for any staining of bile. A Jackson-Pratt drain was placed in the subhepatic space and the abdominal wall and skin were closed following that.

**Figure 1:**
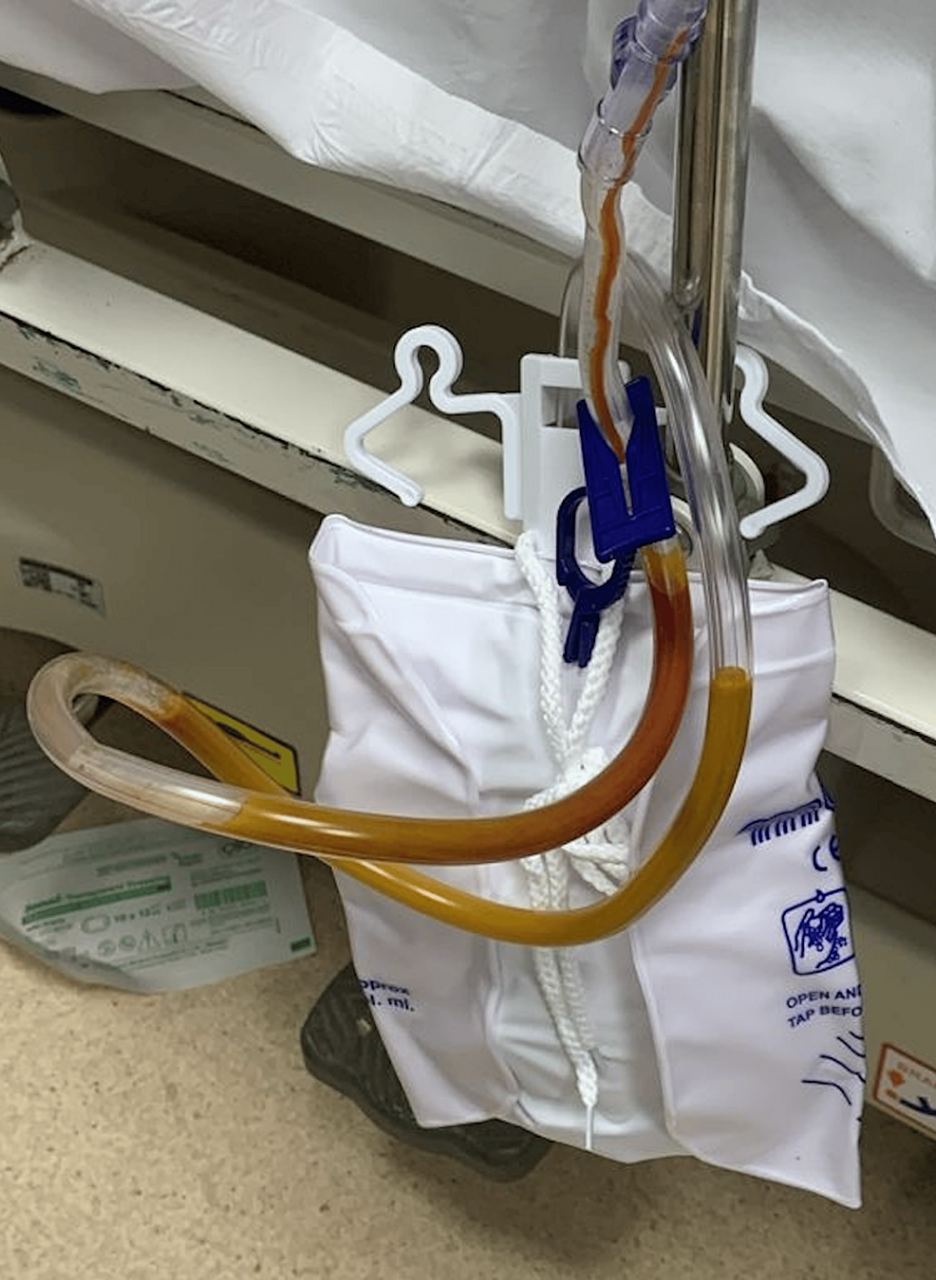
Drain output color

The patient tolerated the procedure well; analgesic and post-operative prophylactic antibiotics were given. Oral intake was resumed later on postoperative day one after the surgery. Postoperative histopathology showed chronic cholecystitis with cholelithiasis. The effluent's initial drain output was around 100 ml/day serosanguinous color. The maximum drain output was 175 ml/day with a serosanguinous color on the second postoperative day; the drain output then decreased day by day. By the sixth day postoperatively, it had significantly decreased to and maintained at 25 mL per day. The patient had good clinical status and laboratory reports were unremarkable. On the seventh postoperative day, the drain output was increased to 200 mL of bile color. The patient's vital signs were stable, with mild abdominal pain. A sample from the drain was sent for culture and showed a growth of Escherichia coli, and an appropriate choice and course of antibiotics were given accordingly. Endoscopic retrograde cholangiopancreatography (ERCP) was done, which showed prior major papilla sphincterotomy, and a bile leak was found from the cystic duct stump, so accordingly, a temporary stent was placed into the common bile duct. After two days, the moderate to minimal amount of drain became milky yellow-whitish in color (Figure 1), with otherwise no abdominal pain and unremarkable clinical and laboratory status. A sample from the drain was sent for triglyceride levels but unfortunately, we didn’t get a result due to lab technical issues.

**Figure 2:**
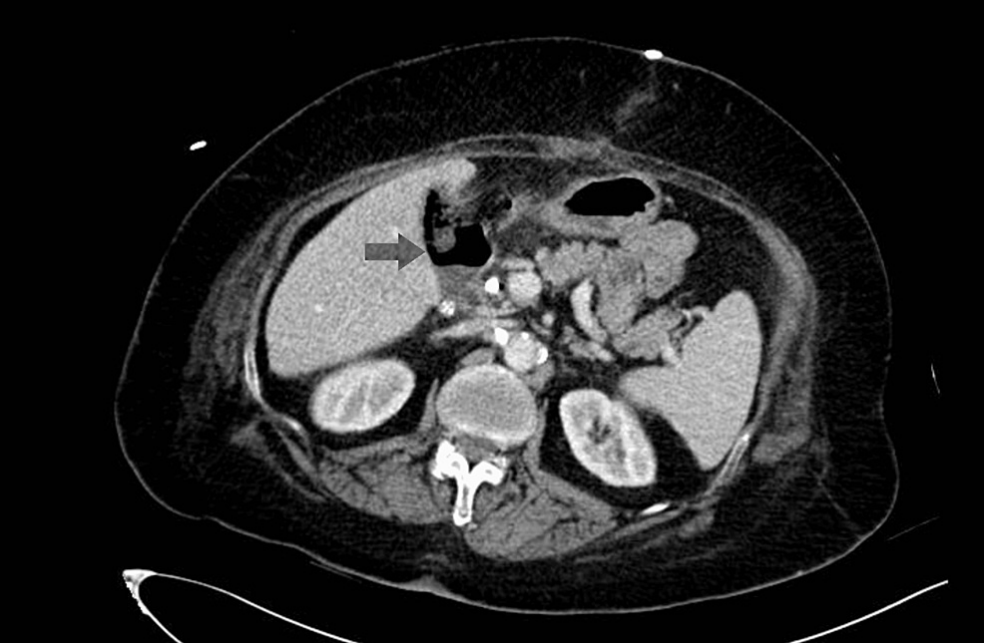
CT abdomen showing the sub-hepatic collection with foci of gas

Abdominal computed tomography (CT) on the thirteenth postoperative day revealed minimal fluid and gas accumulation in the subhepatic space (Figure 2), which was addressed by the surgical drain. With conservative therapy continued, including a low-fat diet with octreotide 100 mcg subcutaneously every 8 hours, the drain output gradually decreased till it became very minimal by the third week postoperatively. The drain was removed and the patient was discharged home in good condition with a stoma bag applied to the removed drain site to follow the amount. After two weeks in the clinic, the patient came with no complaints with a minimal milky amount in the stoma bag, which was removed two weeks later.

## Discussion

Chylous ascites (CA) or chyle leak is a rare form of peritoneal fluid ascites seen with characterizing white milky color formed by accumulated lipid-rich lymph fluids in the abdominal cavity, induced by trauma or lymphatic obstruction [7]. After surgery, chylous ascites appear due to injury to the lymphatic vessels like the thoracic duct, cisterna chyli, or its draining intestinal lymphatic vessels [8]. Chyle leakage following cholecystectomy is extremely rare, with only around seven cases reported in the literature [9-11].

In this case report, we describe our management of a chyle and bile leak in a patient who underwent elective open cholecystectomy. We address this rare complication of post-open cholecystectomy chyle leak with successful conservative management with drain placement, a low-fat diet, and octreotide. The actual cause of our patient's chyle leak remains unclear, however, according to recent articles, chyle leaks following a cholecystectomy are most likely iatrogenic during dissection around the retroperitoneum [12]. Postoperatively, when a patient presents with a chylous leak, from extensive dissection, resections, or cholecystectomies, early recognition and management are advised to avoid further unwanted outcomes. While high-volume chyle leaks are unusual, they have been linked to high mortality reaches up to 70% [13].

The goals of treatment in the literature are to reduce the lymphatic flow and to replace nutrient deficiencies. These goals are achieved mainly through dietary modification and pharmacotherapy. The main two therapeutic diet recommendations are a low-fat diet, a high-protein diet with possible medium-chain triglycerides (MCTs), and total parenteral nutrition (TPN) if needed [9]. The literature presently lacks guidelines for the management of chyle leaks after cholecystectomy due to the rarity of this complication. Prior research has consistently recommended initial conservative treatment and lymphangiography. Patients with persistent high-volume outputs (>500 ml/day) or nutritional impairment should be considered for surgery [14]. To the best of our knowledge, only seven cases reported in the literature, with two requiring surgical interventions where the chyle leak was found laparoscopically on the base of the gallbladder fossa, emanating directly from the parenchyma of the liver bed and controlled using figure-of-eight stitches and application of fibrin glue to help seal the closure [12,14]. One patient had prolonged but successful conservative medical treatment for seven months postoperatively, where the patient was managed with TPN, octreotide, and MCT supplementation, till the drain output decreased to zero [14]. The remaining five cases were treated nonoperatively and included medium-chain triglyceride supplementation and a fat-free or low-fat diet given orally or parenterally, which gradually improved drainage [9,15,16].

In this article, we presented a rare complication of a postoperative chyle leak following an open cholecystectomy. While the patient underwent an ERCP for the treatment of the concurrent bile leak, we were able to successfully manage her chyle leak with a low-fat diet, intravenous antibiotics for the infected fluids with positive body fluid culture, and octreotide. There was no need for further surgical intervention. The chyle leak and bile leak were successfully managed after elective open cholecystectomy.

## Conclusions

A chyle leak is a rare complication seen post cholecystectomy with a poor evidence base to guide the treatment. Conservative management with drainage of the chyle and dietary manipulation might be the preferred approach, with lymphoscintigraphy and surgical interventions reserved for persistent, high-volume leaks or nutritional impairment.
